# The Impact of Leukemia on the Detection of Short Tandem Repeat (STR) Markers

**DOI:** 10.7759/cureus.30954

**Published:** 2022-11-01

**Authors:** Sara F Alharbi, Asim Alamri, Ahmed Elshehawi

**Affiliations:** 1 Biotechnology, Taif University, Taif, SAU; 2 Pediatric Oncology, King Salman Medical City, Medina, SAU

**Keywords:** th01 marker, leukemia, forensic dna analysis, str markers, dna typing

## Abstract

Introduction: Short tandem repeats (STRs) have been used for various identity typing methods worldwide. They have high discrimination power in human identification in forensics, paternity testing, missed personal identification, genetic diseases, and gene regulatory functions. They have also been used to detect and monitor the stability of diseases, including various types of cancer. This study aimed to investigate the impact of leukemia on the detection and stability of STR markers.

Methods: DNA was isolated from 30 participants (15 with chronic myeloid leukemia( CML) and 15 healthy controls) and used to amplify STR markers using specific primers.

Results: We found that the blood of those with leukemia had more 9.3 and 9 alleles at the tyrosine hydroxylase 1 (TH01) marker than the blood of the healthy control samples. The results of this study will help researchers understand leukemia’s effect on the detection and stability of STR markers in leukemic patients compared to healthy individuals.

Conclusion: Our results demonstrate that STR markers could become useful in genetic studies of leukemia cases.

## Introduction

Microsatellites also known as short tandem repeats (STRs), are small DNA sequences (two to six base pairs) with multiple repeats that make up around 3% of the human genome [1]. The number of repeated core units differs substantially among individuals, making them highly distinguishable. The STRs are non-coding sequences [2,3]; however, they can be involved in the regulation of gene expression under various circumstances, resulting in phenotypic effects [4,5]. The use of STRs in forensic science started in 1994 [6].

The STRs have become efficient tools for estimating polymorphism over biochemical and other markers because of several distinct features, including the low required DNA amount and a high number of alleles [7-9]. The STR markers have a wide range of applications for DNA typing in criminal investigations, prenatal diagnoses [10], sibling analyses [11,12], and paternity testing [13,14].

After the publication of the first draft of the human genome [1], numerous studies were undertaken to discover new STR markers in humans [15-23]. Developing and validating STR markers was an essential step toward their use in genotyping. The validated STR markers were used to estimate their frequency in various populations worldwide [24-39].

Detection and estimation of STR markers' frequency is a preliminary step for various populations/subpopulations or different countries prior to their use in various applications. Different countries have conducted such studies including the USA [40,41], Turkey [42], China [43-45], Mexico [46], India [47], and Lybia [48]. For example, the Federal Bureau of Investigation (FBI) selected 13 autosomal STR loci for use in forensic cases and named them the Combined DNA Index System (CODIS). This was reviewed, and seven more markers were added starting in 2017, called the new set CODIS 20, which includes 20 different STR markers: CSF1PO, fibrogen alpha (FGA), tyrosine hydroxylase 1 (TH01), thyroid peroxidase (TPOX), vWA, D3S135, D5S818, D7S820, D8S1179, D13S317, D16S539, D18S51, D21S11, D1S1656, D2S441, D2S1338, D10S1248, D12S391, D19S433, D22S1045 [49].

The STR markers are used to monitor chimerism status in hematopoietic stem cell transplantation (HSCT), an essential process for transplant rejection. Also, it monitors the chimerism percentage and stability of STR markers in leukemic patients after transplantation [50-52].

The instability of STR markers in cancerous tissues could occur because of defects in the DNA repair pathways and the accumulation of alterations in microsatellite loci. The STR markers exhibit instability in various cancers, including lung [53], papillary thyroid [54 ], esophageal [55], and leukemia [56]. Nearly 30 Mendelian human disorders have STR expansions as the underlying DNA mutation [57]. Moreover, for forensic purposes, STRs study the parental sources of common chromosomal aneuploidies. For example, trisomies can be accurately detected, and their parental sources can be traced with 92.54% accuracy [58].

In Saudi Arabia, few studies have been conducted on STR markers to detect allele variations in subpopulations as genetic diversity of forensic STR markers [59-64]. The overall prevalence of leukemia was estimated as 7.6% in males and 4.4% in females in the Saudi population [65]. To the best of our knowledge, no studies have been conducted on the detection of STR markers in specific groups of patients. Therefore, the focus of this study is to investigate the detection and stability of STR markers in leukemic cases.

## Materials and methods

Study design, ethics statement, and human participants 

The current research is a bioethical case-control study. We obtained the approval of the Dean of Scientific Research at Taif University in the Kingdom of Saudi Arabia as well as that of the King Salman Medical City Institutional Review Board (approval no. H-03-M-11). Participants signed consent forms before blood samples were collected at the King Fahad Hospital's Department of Oncology and Hematology in Medina, SAU. Furthermore, the control samples selection criteria are based on those who donate blood voluntarily, are healthy, and have normal complete blood counts while the leukemia case selection criteria are based on diagnostic tests.

Case selection, sample collection, and sample size

A total of 30 participants (15 with chronic myeloid leukemia( CML) and 15 healthy controls) were included in the study. All participants were aged 35 to 61. Of a total of 15 CML cases, only three (participants 1, 2, and 5) had undergone chemotherapy treatment. The analyses were performed on blood samples placed on Flinders Technology Associates (FTA) cards. 

DNA extraction

The DNA was extracted from blood with a PrepFiler Express™ Forensic DNA Extraction Kit, which was used according to the manufacturer’s instructions (Thermo Fisher Scientific, Waltham, MA, USA). The extracted DNA samples were fluorometrically quantified with a Quantifiler™ Human DNA Quantification Kit (Thermo Fisher Scientific, Waltham, MA, USA), which was used according to the manufacturer’s instructions.

DNA amplification 

Amplification of the 16 STR loci (D8S1179, D21S11, D7S820, CSF1PO, D3S1358, TH01, D13S317, D16S539, D2S1338, D19S433, vWA, TPOX, D18S51, acute myeloid leukemia (AML), D5S818, and FGA was performed with an AmpFLSTR™ Identifiler™ Kit Plus PCR Reagents (Applied Biosystems, Waltham, MA, USA), which was used according to the manufacturer’s instructions. Polymerase chain reaction (PCR) was carried out with a Gene-Amp® PCR 9700 thermal cycler (Applied Biosystems; Thermo Fisher); initial incubation was at 95 °C for 11 min, followed by 30 cycles of denaturation (94 °C, 20 s), annealing (59 °C, 2 min), and extension (72 °C, 1 min).

STR genotyping

Capillary electrophoresis was performed using POP-4™ Polymer (Applied Biosystems) in an ABI Prism 3500 Genetic Analyzer (Thermo Fisher) under default conditions (i.e., injection time, 15 s; run time, 1500 s) with the following components per sample: 12 μL Hi-Di™ Formamide (Thermo Fisher), 0.5 μL 500 LIZ™ Size Standard (Applied Biosystems) and 0.5μL PCR product. Prior to fragment analysis, this mixture was heat-denatured for seven minutes at 95 °C and snap-cooled on ice for another three minutes, after which it was transferred onto a plate and placed in the genetic analyzer.

Genetic evaluation

Specimens were evaluated using the specialized software, GeneMapper® IDX version 1.4 (Thermo Fisher Scientific). The analytical and stochastic thresholds were set to 50 RFU and 200 RFU, respectively. The profiles obtained from the blood samples of the leukemic patients were then compared to those from the healthy individuals to observe the difference in genetic information.

Statistical analysis

Data analysis was performed using Statistical Package for Social Sciences (SPSS) version 26 (IBM Corp., Armonk, NY, USA). A two-way contingency table was created to investigate whether associations existed between different alleles and leukemia. Two alleles (allele 9 and allele 9.3) were used as predictors of leukemia. Because of the small sample size, the use of contingency tables larger than 2 × 2, and the presence of 2 cells with an expected count < 5, the Fisher-Freeman-Halton exact test was performed. The odds ratio (OR) and confidence interval (CI) were also calculated.

## Results

Thirty samples collected from 15 patients with leukemia and 15 healthy individuals were used in this study to investigate the stability and detection of 15 somatic STR markers and the amelogenin (AML) marker. All markers were successfully detected in all participants. Three markers-D21S11, TH01, and D19S433-showed microvariants (see Table 1).

**Table 1:** Summary of the volunteers’ STR genotypes STR: Short tandem repeat, TH01: Tyrosine hydroxylase 1, TPOX: Thyroid peroxidase, AML: Acute myeloid leukemia, FGA: Fibrogen alpha

Sample #	D8S1179	D21S11	D7S820	CSF1PO	D3S1358	TH01	D13S317	D16S539	D2S1338	D19S433	vWA	TPOX	D18S51	AML	D5S818	FGA
1	10,13	29,29	8,8	10,13	16,17	7,9	8,8	12,12	19,20	13,14.2	16,17	8,11	14,15	XY	9,10	23,25
2	13,14	29,32.2	10,10	11,12	15,17	8,9	8,11	9,11	20,20	15,16	18,18	9,10	12,17	XX	9,13	19,23
3	12,16	29,32.2	8,9	10,13	17,17	6,9.3	9,11	9,13	18,22	13,15.2	16,18	8,8	16,17	XY	12,12	19,23
4	13,13	29,29	8,10	10,11	16,16	8,9	8,11	10,11	17,20	15.2,16	15,20	8,12	11,18	XY	12,12	24,25
5	10,13	29,29	8,8	10,13	16,17	7,9	8,8	12,12	19,20	13,14.2	16,17	8,11	14,15	XY	9,10	23,25
6	16,16	28,29	10,10	10,12	15,17	6,9	8,12	10,12	17,24	12,14	14,15	8,11	14,17	XX	12,13	19,24
7	11,13	29,32.2	11,12	10,11	16,18	6,9	8,11	11,13	19,20	15.2,16	14,17	9,9	12,14	XX	11,14	24,24
8	12,14	28,30	10,11	10,13	16,16	7,9.3	12,12	9,12	16,18	15,15.2	15,17	8,8	13,14	XX	12,12	21,24
9	14,15	30,32.2	8,11	11,12	16,17	7,9	8,11	11,12	17,20	13.2,15	16,17	8,8	13,15	XX	11,12	22,24
10	11,13	28,29	8,10	12,13	16,17	6,9.3	10,14	12,13	16,17	13,14	17,18	8,9	13,17	XY	9,12	21,25
11	13,15	29,30	10,11	9,12	15,16	9.3,9.3	11,12	11,13	16,18	12.2,14	17,17	8,8	13,13	XY	11,12	24,26
12	11,14	30,31	11,12	11,11	15,17	9.3,10	8,12	11,11	16,19	14,14.2	15,16	8,9	12,12	XY	10,12	21,22
13	13,14	29,31.2	10,12	10,12	15,15	6,9	11,12	9,12	17,23	13,15.2	15,17	10,11	13,13	XX	12,13	19,24
14	14,15	30,31	11,13	11,12	15,15	6,9	12,13	9,12	16,20	14,15	18,19	8,11	13,15	XX	11,12	21,24
15	11,11	28,28	11,12	11,11	16,17	9,9	11,11	10,11	19,19	1214	14,17	9,9	13,15	XX	12,13	24,25
1C	10,11	30,32.2	11,11	10,11	17,17	7,7	8,12	11,12	18,20	15,15.2	16,18	9,11	16,16	XY	10,11	13,13
2C	10,13	30,32.2	10,11	11,13	17,18	6,7	11,12	11,12	20,21	13,15.2	16,18	9,11	16,16	XX	11,13	23,23
3C	10,13	30,31.2	8,10	11,11	17,18	6,7	8,12	11,12	18,21	12,13	16,18	9,11	12,16	XY	10,11	23,23.2
4C	14,15	29,31	9,10	11,13	16,17	7,7	12,13	11,11	22,22	13,14	17,20	10,11	14,17	XX	8,10	22,24
5C	10,11	30,32.2	11,11	10,12	15,17	7,7	8,12	11,12	18,20	15,15.2	16,18	9,11	16,16	XY	10,11	21,23
6C	13,15	30,32.2	9,11	10,12	16,17	6,7	11,13	8,13	17,18	14,16	19,20	8,9	16.2,17	XX	12,13	23,23
7C	12,15	28,31	10,10	10,10	18,18	7,8	12,12	9,9	17,23	13,13	17,18	8,8	16,18	XX	12,12	23,24
8C	14,16	28,31.2	8,12	11,12	15,16	6,9.3	9,11	11,13	23,24	14,15	17,18	9,11	14,16	XX	11,12	20,25
9C	11,14	30,30	11,12	10,11	15,17	7,9	11,12	11,11	17,20	14,15.2	17,18	8,9	12,15	XY	11,12	22,22
10C	13,16	30,31.2	12,12	12,12	15,17	6,7	11,11	11,11	20,22	14,15	17,18	8,9	14,15	XY	10,12	21,22
11C	12,13	30,31.2	10,10	10,10	15,16	7,9	12,14	9,10	20,21	13.2,14	16,18	8,8	16,18	XY	12,12	19,23
12C	14,14	31.2,32.2	8,12	10,12	15,16	7,9	8,11	10,11	17,18	12,13	16,19	8,10	13,13	XY	12,13	21,24
13C	10,14	30,31.2	8,9	10,11	15,17	7,8	10,13	11,12	25,25	12,14.2	18,18	8,8	12,12	XX	11,12	20,22
14C	12,14	32.2,32.2	10,12	10,12	15,18	6,9	11,13	11,11	18,22	15,15	15,15	8,9	12,16	XY	12,13	19,21
15C	10,12	29,30	10,11	11,11	17,18	7,9	8,11	9,11	21,25	14,15.2	18,18	8,9	13,13	XY	12,13	24,25

The proportion of alleles 9 in patients with leukemia was significantly higher than in controls. A significant difference in allele distribution between cases and controls was noticed (χ2 = 18.567, df = 2, p < 0.000). Table 2 shows that 77% of leukemia patients were found to have allele 9, compared to 23.1% of control cases, indicating a strong significant association between them (χ2= 6.652, df = 1, 2, odds ratio = 8, 95% CI = 1.522 - 42.042, P-value =0.025). However, no significant association was observed between allele 9.3 and leukemia.

**Table 2:** Differential distribution of genotypes in leukemia cases and controls. OR: Odds ratio, CI: Confidence interval, df: Degree of freedom

	Leukemia Cases		Control cases				
Genotype	n	(% within exposure)	n	(% within exposure)	OR	95% CI	Test of Significant
9 allele	10	77.0%	5	23.1%	8	1.522 – 42.042	χ2= 6.652, df = 1; P-value =0.025
9.3 allele	5	83.3%	1	16.7%	7	0.705 – 69.490	χ2= 3.333, df = 1; P-value =0.169

**Figure 1:**
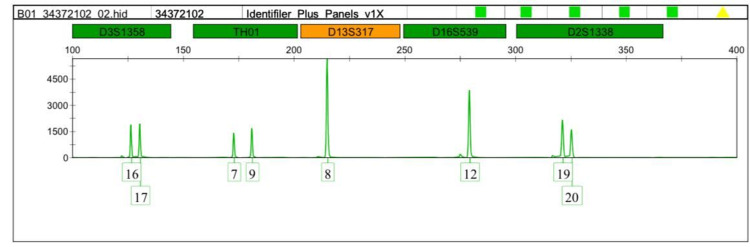
DNA profiling of the leukemia case (volunteer no.1) TH01:  Tyrosine hydroxylase 1

**Figure 2:**
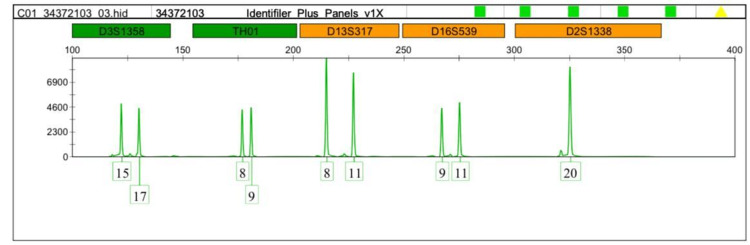
DNA profiling of the leukemia case (volunteer no.2) TH01:  Tyrosine hydroxylase 1

**Figure 3:**
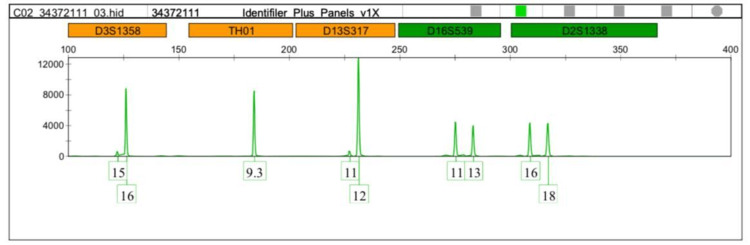
DNA profiling of the leukemia case (volunteer no.11) TH01:  Tyrosine hydroxylase 1

**Figure 4:**
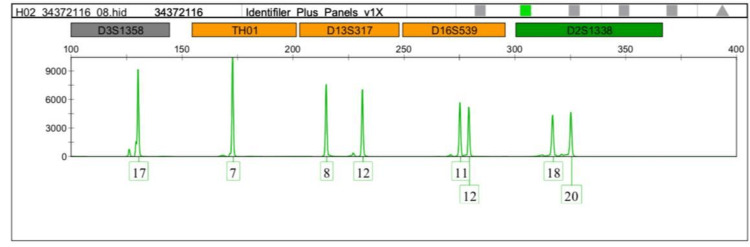
DNA profiling of the control case (volunteer no.1) TH01:  Tyrosine hydroxylase 1

**Figure 5:**
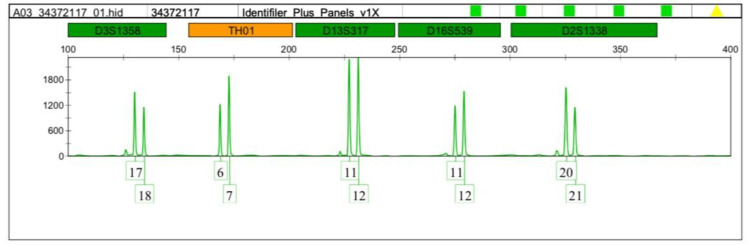
DNA profiling of the control case (volunteer no.2) TH01:  Tyrosine hydroxylase 1

Interestingly, the TH01-9 and the micro variant 9.3 were detected more in the leukemia cases. Therefore we focused on comparing allele 9 and the microvariant allele 9.3 between normal and leukemic cases as shown in Figures 1-5.

## Discussion

In recent years, reports of the links between genetic markers in populations and diseases have increased. Several studies have used forensic STRs as genetic markers to look for cancer-related alleles. Furthermore, many studies have reported a strong association between TH01 polymorphism and its related alleles in several diseases compared to control groups. 

The present study, even with its small sample size, is in concordance with research [66]; Qi et al. conducted a study to analyze the feasibility of screening for lung and liver cancer predispositions using genetic markers rather than related genes. Their findings revealed a statistically significant difference between the D18S51-20 allele and lung cancer-related allele, as well as between the D21S11-30.2 and D6S1043-18 alleles and liver cancer-related alleles. These findings show that CODIS indicators can predict a person’s propensity for cancer. In another study, young individuals with gastric cancer were shown to have had a significantly high frequency of two sets of alleles, D2S1338-23 and D6S1043-11, and D8S1179-16 and D5S818-13 [67].

According to the GenBank top strand nomenclature, TH01 is situated inside the first intron of the human tyrosine hydroxylase gene, and the chromosomal location is on 11p15.5. Frequently defined by the repeat patterns [AATG]n or [TCAT]n as allele 9 [AATG]9 while allele 9.3 [AATG]6ATG[AATG]3 [68,69]. The biological mechanism of TH is responsible for rate-limiting during the production of catecholamines (e.g., dopamine, epinephrine, and norepinephrine), which are neurotransmitters and hormones that help maintain the body’s homeostasis [70].

Researchers have found an important link between changes in TH expression and the development of neurological, psychiatric, and cardiovascular diseases [70,71]. Wei et al. discovered that among a group of unrelated healthy adults, those with the TH01-9 allele had the highest serum norepinephrine levels, whereas those with the TH01-7 allele had the lowest levels [72].

Our DNA profiling showed a strong link between TH01 (alleles 9) and the presence of leukemia. Despite the limited sample size, this finding confirms the association between the allelic configuration of STR markers and CML [73]. Additionally, These results are supported by previous studies that demonstrated strong relationships between TH01 and the predisposition to several diseases, such as malaria [74], thrombosis [75], hypertension [76], Parkinson’s disease [77], and sudden infant death syndrome (SIDS) [78,79].

## Conclusions

This study presented the genotype of leukemia cases as a piece of significant evidence. The predominance of allele‐9 at the TH01 locus (AATG repeat unit) in leukemia cases indicates an association with leukemia. Furthermore, the TH01 microsatellite combined with alleles 9 is a good biomarker candidate for leukemia. While microvariant allele 9.3 was found in leukemia cases, it was not significant due to the small sample size. However, STR markers could become useful in genetic studies of leukemia cases. Finally, forensic STR markers may provide more information than just identity.
